# microRNAs in the Regulation of Adipogenesis and Obesity

**DOI:** 10.2174/156652411795677990

**Published:** 2011-06

**Authors:** R.A McGregor, M.S Choi

**Affiliations:** Center for Food & Nutritional Genomics Research, Department of Food Science and Nutrition, Kyungpook National University, Daegu, Republic of Korea

**Keywords:** Adipocytes, adipogenesis, biomarkers, microRNAs, miR-27, miR-519d, obesity.

## Abstract

Worldwide obesity is a growing health problem, associated with increased risk of chronic disease. Understanding the molecular basis of adipogenesis and fat cell development in obesity is essential to identify new biomarkers and therapeutic targets for the development of anti-obesity drugs. microRNAs (miRNAs) appear to play regulatory roles in many biological processes associated with obesity, including adipocyte differentiation, insulin action and fat metabolism. Recent studies show miRNAs are dysregulated in obese adipose tissue. During adipogenesis miRNAs can accelerate or inhibit adipocyte differentiation and hence regulate fat cell development. In addition miRNAs may regulate adipogenic lineage commitment in multipotent stem cells and hence govern fat cell numbers. Recent findings suggest miR-519d may be associated with human obesity, but larger case-control studies are needed. Few miRNA targets have been experimentally validated in adipocytes but interestingly both miR-27 and miR-519d target PPAR family members, which are well established regulators of fat cell development. In this review recent advances in our understanding of the role of miRNAs in fat cell development and obesity are discussed. The potential of miRNA based therapeutics targeting obesity is highlighted as well as recommendations for future research which could lead to a breakthrough in the treatment of obesity.

## INTRODUCTION

Within the next five years the World Health Organization projections indicate approximately 2.3 billion adults will be overweight and more than 700 million adults will be obese. Obesity is a major risk factor for chronic diseases such as cardiovascular disease and Type 2 diabetes [1,2]. In recent years despite advances in understanding the molecular basis of obesity, anti-obesity drugs lack physiological specificity and have side-effects [3]. In the past decade the discovery of non-coding microRNAs (miRNAs) which can post-transcriptionally regulate thousands of genes has generated enormous research interest [4,5]. Now miRNA biomarkers have been found in many chronic diseases such as cancer, cardiovascular disease and Type 2 diabetes [4, 6-8]. Recently, evidence of miRNA dysregulation has been reported in human obesity [9]. In future miRNA biomarkers may assist in the early diagnosis of chronic diseases and also provide new therapeutic targets [10]. With recent advances it is a real possibility miRNA signatures from patient’s tissue or plasma could be used for personalized diagnosis and treatment [6,10]. Hence it is a pertinent time to review our current understanding of the role of miRNAs in adipogenic differentiation and obesity. Firstly, this review will provide a brief overview of the association between adipocyte biology and obesity. Secondly, our current understanding of miRNA biogenesis, regulation and function are summarized. Thirdly, recent advances in the role of miRNAs in fat cell development and obesity are discussed based on mouse and human studies. Furthermore, the impact of extracellular factors such as inflammatory cytokines on adipocyte miRNAs is considered. In closing the potential of miRNA based therapeutics for anti-obesity treatments will be highlighted along with recommendations for future research.

## OBESITY

Obesity is characterized by increased fat mass and energy storage in adipose tissue [2]. Increases in fat-mass can be achieved by increases in the size of adipocytes (adipocyte hypertrophy), or expanding the numbers of adipocytes (adipocyte hyperplasia) [11]. In addition, obesity is strongly associated with inflammation and insulin resistance [12], although not always [13]. Larger fat cells are closely linked to greater fat mass and the production of inflammatory cytokines [11]. Nevertheless, alterations in adipocyte turnover rate, differentiation and apoptosis could all contribute to changes in fat mass underlying obesity. However, recent findings suggest the turnover rate of pre-adipocytes in humans is very low, amounting to 10% self-renewal every year [14]. Other studies have suggested pre-adipocyte differentiation may be impaired in obese humans [15-17]. Larger fat cells attract macrophages leading to adipocyte necrosis and release of fatty acids into circulation which contributes to excess fat deposition in the liver [11]. One possible avenue to reduce fat mass is to therapeutically regulate adipocyte differentiation, but a better understanding of the pathways controlling adipogenesis is needed.

In recent years, there has been a rapidly growing interest in the role of miRNAs in fat cell development and obesity [18,19]. Studies have showed miRNA expression in pre-adipocytes is altered during fat cell development and in obesity (Table **1**). Understanding the role miRNAs play in the proliferation and differentiation of adipocytes during fat cell development could provide new therapeutic targets for anti-obesity drugs. In addition, identifying miRNAs dysregulated during the development of obesity, could provide early obesity biomarkers for clinical diagnosis.

### miRNAs

miRNAs are a class of short non-coding RNAs (19-22 nucleotides) involved in the post-transcriptional regulation of genes [20]. miRNAs can bind to complementary target sites in mRNA genes which can cause translation repression or cause cleavage, deadenylation and degradation of target mRNA genes [21]. miRNAs may regulate over one third of protein-coding genes, for example, overexpression of specific miRNAs in HeLa cells revealed hundreds of transcripts are modulated, but the down-regulated transcripts were more likely to contain conserved miRNA binding sites [22]. Further studies have shown widespread regulation of protein levels by miRNAs in cellular and animal models [23,24], yet the true magnitude of post-transcriptional regulation by miRNAs in humans is unknown. Computational predictions suggest 45000 putative miRNA binding sites may exist in human protein coding genes [25]. However, many of the putative miRNA binding sites remain to be experimentally validated.

## miRNA BIOGENESIS AND PROCESSING

Understanding of miRNA biogenesis and regulation has advanced rapidly in recent years and has been reviewed elsewhere [20,26,27]. miRNAs are transcribed from intronic or intergenic regions, while some miRNAs are encoded within protein coding genes [20]. The main steps in the miRNA processing pathway are illustrated in Fig. (**1**). miRNAs are transcribed as longer primary miRNA (pri-miRNA) transcripts by RNA polymerase II and processed into functional mature miRNAs by miRNA processing proteins [26]. In the nucleus pri-miRNA sequences are cleaved by a microprocessor complex including the RNAase III enzyme Drosha and DGCR8, which results in shorter 70-80 nucleotide precursor miRNAs (Pre-miRNA) [28]. Precursor miRNAs are rapidly exported from the nucleus to the cytoplasm mainly *via *Exportin5 which is a GTP dependent nuclear transport protein [29]. In the cytoplasm pre-miRNAs are processed by the Dicer, leaving an unstable miRNA duplex which unwinds, the 5’ guide strand containing the mature miRNA sequence is incorporated into a ribonucleotide silencing complex (RISC) while the 3’ passenger strand is rapidly degraded [30].

The RISC consist of multiple proteins, many of which remain to be fully characterized [31]. Argonaute proteins contain multiple GW182 binding sites which facilitate miRNA localization to cytoplasmic P-bodies [27]. The mammalian homologue of GW182 is TNRC5 which regulates miRNA processing in human cells. P-bodies contain deadenylases and decapping enzymes such as CAF1 and PABP which can cause adenylation and degradation of target mRNAs [32]. P-bodies can also act as temporary storage depots for mRNAs and therefore reduce the translation of mRNAs into proteins [32].

miRNAs are generally reported to have long half-lives, but little is known about the mechanism which regulates miRNA degradation in cells. XRN-2 which is a 5′→3′ exoribonuclease has been reported to mediate the degradation of mature miRNAs in *Caenorhabditis elegans*, but no studies have examined miRNA degradation in human cells [33-35]. Elucidating the mechanism leading to miRNA degradation could explain the downregulation of miRNAs in some chronic diseases such as Type 2 diabetes and provide new therapeutic targets to restore miRNA homeostasis [36].

## REGULATION OF miRNA PROCESSING

miRNA processing may be regulated by multiple factors [27]. Some miRNAs are processed efficiently, while other miRNAs show large discrepancies between pre- and mature-miRNA abundance [37]. Alterations in miRNA processing proteins Drosha and Dicer can cause widespread decreases in mature miRNAs and have been associated with cancer [38]. However, recent advances are revealing more sensitive and selective factors exist which can regulate miRNA processing [27].

miRNAs can be transcriptionally regulated by proteins binding upstream of miRNA genes [26]. For example SMAD proteins are reported to bind to the genomic region encoding miR-21 and suppress miR-21 transcription [39]. miRNAs can also be post-transcriptionally regulated by RNA-binding proteins [26]. For example, hnRNP-A1 (heterogeneous nuclear ribonucleoprotein A1) can bind to pre-miR-18a and block further processing by Drosha [40]. Furthermore, in embryonic stem cells LIN28 protein can bind to a conserved region in the miRNA stem loop encoding let-7, which leads to inhibition of Drosha activity, thus preventing embryonic stem cell differentiation [41]. Intriguingly, 14% of all pri-miRNAs are reported to have highly conserved stem loops, which suggests these may act as landing pads for RNA-binding proteins [42] and hence provide a further regulatory layer in the miRNA processing pathway.

RNA binding proteins can also disrupt miRNA target sites for example DND1 (Dead end 1) can bind to miRNA target sites in the 3’UTR hence blocking miRNA binding and miRNA-mediated translational repression [43]. To date existing evidence suggests mature miRNA expression is tightly regulated by multiple factors including RNA-binding proteins and miRNA processing proteins [27], but so far there have been no studies on the regulation of the miRNA biogenesis pathway in adipocytes.

## IDENTIFICATION OF miRNA TARGETS

miRNAs are important post-transcriptional regulators of gene expression because an individual miRNA can target hundreds of genes [20]. Therefore, several miRNAs can act combinatorially to regulate a network of target genes. miRNA target genes can be computationally predicted based on several factors [20]. Mature miRNAs contain a seed region between nucleotide 2-7 which can bind to complementary sequences in the 3’UTRs of target genes [44]. Perfect complementarity between miRNA seed sequence and 3’UTR increases the likelihood of target repression, but imperfect seed matches can also lead to target repression [44]. miRNA target prediction algorithms are widely available, which can predict miRNA targets across different species [45]. The most established and widely used miRNA target prediction algorithms are TargetScan [44], PicTar [46] and Miranda [47]. Each uses a different set of rules to identify and evaluate the efficacy of a miRNA target using a unique scoring system [45]. Evaluating the utility of each prediction algorithm is difficult as there is no comprehensive set of experimentally validated targets [20]. Furthermore, it is possible miRNAs which normally repress translation can also activate translation of target mRNAs depending on cell cycle [48]. Currently, there is still debate whether miRNAs predominantly act *via *translational repression or mRNA degradation of targets, which has important implications for designing studies to validate predicted miRNA targets [20]. Several studies suggest miRNAs may predominantly function *via *mRNA cleavage and hence could be detectable by genome-wide microarrays [22-24]. Unfortunately there are no universally accepted criteria for validating miRNA targets [49].

## EXPERIMENTAL VALIDATION OF miRNA TARGETS

Experimental validation of miRNA targets involves measuring changes in predicted target proteins in response to ectopic miRNA expression or miRNA knockdown. In addition, it is important to demonstrate a miRNA can specifically bind to its predicted target gene. Typically, miRNA: mRNA target binding can be demonstrated by transfecting a miRNA inhibitor or miRNA mimic alongside a Luciferase reporter containing the 3’UTR of the predicted target mRNA [49]. Novel approaches to measuring global changes in miRNA targets are possible by microarray profiling of RNA bound to immunoprecipitated Argonaute proteins [50]. Alternatively, global target protein changes in response to cellular miRNA manipulation can be tracked using stable isotope labeling with amino acids in cell culture (SILAC) techniques [23]. Whichever approach is used, the consensus among miRNA researchers is that experimental validation of predicted miRNA targets remains necessary to confirm the functional relationship between a miRNA and its target mRNA [20].

## miRNA MUTANTS CHARACTERIZED BY AN OBESE PHENOTYPE

The first evidence of a role for miRNAs in fat cell metabolism and obesity came from genetic screens in Drosophila [51]. miR-14 deletion in Drosophila caused the development of a phenotype characterized by apoptosis, enlarged adipocyte lipid droplets, elevated triglycerides and diacylglycerol [51]. These findings suggested miR-14 plays a role in fat metabolism and cell death. A later study reported mutant miR-278 Drosophila were characterized by a lean phenotype with reduced adiposity [52]. The miR-278 mutants also had high circulating sugar levels despite elevated insulin production, which suggested lack of miR-278 may lead to insulin resistance [52]. Ectopic expression of the EX (Expanded) gene which can act as a tumor suppressor and also harbors a putative miR-278 target site resulted in a similar phenotype to the miR-278 mutants [52]. Although these studies implicated miRNAs in fat metabolism, cell death and insulin resistance, neither miR-278 nor miR-14 have known human homologs and therefore have not been subsequently examined in obese humans.

## miRNAs AND ADIPOCYTE DIFFERENTIATION

**Fig. (2):**
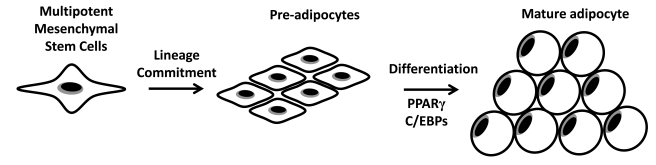
Fat cell development: Multipotent mesenchymal stem cells can develop into lineage committed pre-adipocytes. PPAR and C/EBP transcription factors co-ordinate adipogenic gene expression during terminal differentiation into lipid storing mature adipocytes.

In adipose tissue increased fat mass associated with obesity is characterized by both increased adipocyte size and/or increased adipocyte number [11]. The process of adipogenesis largely determines the number of adipocytes in body fat depots. Pre-adipocytes proliferate and can develop into mature adipocytes upon growth arrest, clonal expansion and terminal differentiation [53]. Mature adipocytes are insulin sensitive and can store large amounts of lipids [53]. Another source of pre-adipocytes is multipotent MSCs (Fig. **2**). These multipotent MSCs can develop into lineage committed pre-adipocytes upon hormonal stimulation and differentiate into mature lipid storing adipocytes [54]. Currently the role of multipotent MSCs in the development of human obesity is unclear.

To date studies have profiled miRNA expression during adipogenic differentiation predominantly in the mouse 3T3-L1 cell-line [55-59]. Studies have used miRNA arrays in combination with validation by Northern blot or RT-qPCR, but with no universally accepted miRNA array platform there is little agreement between studies on candidate miRNAs (Table **2**) [18]. Some miRNAs appear to be negative regulators of adipocytes differentiation while some miRNAs are capable of accelerating adipocyte differentiation [18,19].

## miRNAs CAN ACCELERATE ADIPOCYTE DIFFERENTIATION

A screening study of adipogenic miRNAs using ASO (anti-sense oligonucleotides) directed at a large panel of miRNAs in human primary sub-cutaneous pre-adipocytes revealed inhibition of miR-9* and miR-143 suppressed the adipogenic markers GLUT4, HSL, FABPaP2, PPAR-γ2 and triglyceride accumulation [60]. miR-143 is also reported to be strongly induced during pre-adipocyte 3T3-L1 differentiation [55,61,58] and human pre-adipocyte differentiation [60]. Ectopic miR-143 expression during differentiation of 3T3-L1 pre-adipocytes resulted in twice the level of triglyceride accumulation early during differentiation [55]. There have been few reports of experimental validation of miR-143 targets in adipocytes.

One study demonstrated ERK5 (Extracellular-signal-regulated kinase 5) is targeted by miR-143 in human pre-adipocytes [60]. The role of ERK5 in adipocyte differentiation is not clear, although it has been suggested ERK5 suppression may be involved in fine-tuning the MAPK (mitogen activated protein kinase) pathway to maintain the differentiated state [55]. In a high-fat diet model of obesity miR-143 was found to be upregulated in mesenteric adipose tissue of mice, yet only a modest downregulation of miR-143 target protein ERK5 was observed *in-vivo *[62]. If miR-143 is pro-adipogenic then developing miR-143 inhibitors may be a useful approach to slow down adipocyte differentiation and lipid droplet formation. Ideally studies are needed to determine whether triglyceride accumulation can be altered by miR-143 inhibition in mature adipocytes.

Several other miRNAs have been identified which can accelerate adipocyte differentiation [55, 56, 59, 61, 63-65]. miR-103 is reported to be upregulated during differentiation of human pre-adipocytes [60]. When miR-103 is overexpressed in the presence of adipogenic stimuli, adipogenesis accelerates, as shown by increased triglyceride accumulation and adipogenic gene expression [55]. *In-vivo *miR-103 was reported to be downregulated in mature adipocytes from obese mice [55], although studies in obese human  adipose tissue show miR-103 is upregulated [9, 66]. The lack of consistency between studies is most likely due to differences in fat depots between mice and humans. It appears more experimental work is needed to establish the role of miR-103 in adipogenesis and obesity.

The miR-17/92 cluster is reported to be significantly upregulated during 3T3-L1 differentiation [57]. The miR-17-92 cluster consists of five members, miR-17-5p, miR-17-3p, miR-18, miR-19b and miR-20, of which only miR-20 was previously reported to be upregulated in mature differentiated adipocytes [60]. The miR-17-92 cluster appeared to target Rb2 and p130, which are both negative regulators of differentiation. Overexpression of the miR-17-92 cluster was reported to translationally repress Rb2 and p130 resulting in rapid adipocyte differentiation [57].

Clonal expansion in 3T3-L1 pre-adipocytes is reported to be inhibited by Let-7 [67], although Let-7 increases during later adipocyte differentiation. Clonal expansion usually follows growth arrest and involves the replication of pre-adipocytes prior to terminal differentiation. Ectopic expression of Let-7 markedly reduces HMGA2 which is a high mobility group (HMG) protein. HMG proteins function as architectural factors and are essential components of the enhancesome. In mice lacking HMGA2 adipose tissue is significantly reduced [67]. Furthermore, overexpression of HMGA2 in obese leptin deficient mice reduces adipose tissue mass [67]. To date elevated HMGA protein has not been reported in human obese adipose tissue.

**Fig. (3):**
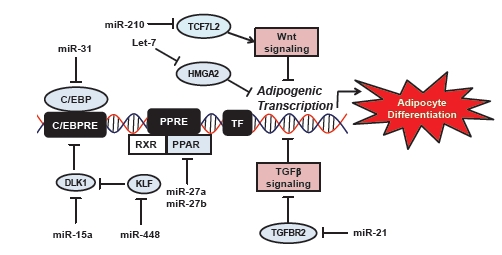
Mammalian miRNAs regulate target genes during adipogenesis.

Another adipogenic miRNA to emerge recently is miR-210 which has been demonstrated to regulate TCF712 (Fig. **3**), which is a key transcription factor modulating components of WNT signaling [59]. Overexpression of miR-210 in 3T3-L1 cells is reported to stimulate adipocyte hypertrophy and lipid droplet formation [59]. The upregulation of miR-210 during adipogenesis is in concordance with a suppression of genes encoding proteins in the WNT signaling pathway [59].

## miRNAs CAN SUPPRESS ADIPOCYTE DIFFERENTIATION

Some studies have focused on miRNAs which appear to act as negative regulators of adipocyte differentiation (Fig. **3**). For example, overexpression of miR-27a in 3T3-L1 pre-adipocytes suppresses PPARγ expression and adipocyte differentiation [68]. The 3’UTR of PPARγ harbors a putative miRNA binding site, which has been shown to specifically bind to miR-27a using a luciferase reporter assay [68]. Another family member, miR-27b is also downregulated during adipocyte differentiation. MiR-27b can also bind to the 3’UTR of PPARγ and repress PPARγ protein levels [69]. However, transfection of miR-27b two days after adipogenic stimulation was not sufficient to repress PPARγ protein in 3T3-L1 adipocytes [68]. Interestingly, in mature adipocytes from obese mice lower miR-27a expression has been found compared to lean mice, indicating miR-27a downregulation may be necessary for adipocyte hypertrophy [70]. These studies suggest the miR-27 family could be a useful anti-adipogenic target. Potentially miR-27a mimics could be used to regulate pre-adipocyte proliferation. However, suppressing mature adipocyte differentiation and the concomitant reduction of lipid storage capacity may lead to lipids being stored in liver and skeletal muscle with detrimental side-effects such as insulin resistance or steatosis [11].

Another study reported miR-448 is a potential inhibitor of adipogenesis [71]. miR-448 is encoded within the intron of HTR2C, a serotonin receptor which is upregulated during 3T3-L1 adipocyte differentiation [71]. Kruppel-like factor 5 (KLF5) contains a putative miR-448 binding site which was experimentally validated using a luciferase reporter assay. Overexpression of miR-448 in pre-adipocytes suppresses KLF5, triglyceride accumulation and adipogenic gene expression thus suggesting miR-448 is a negative regulator of adipocyte differentiation [71].

Inhibition of miR-15a appears to reduce pre-adipocyte size while promoting adipocyte proliferation [72]. In preadipocytes miR-15a has been shown to target Delta homologue 1 (DLK1) at mRNA and protein level [72]. Inhibition of miR-15a in pre-adipocytes resulted in a decrease in cell size along with an increase in cell number [72]. Despite many published miRNA profiling studies in 3T3-L1 pre-adipocytes, mouse and human pre-adipocytes only one other study reported miR-15a was upregulated during pre-adipocyte differentiation [61]. More studies are needed to fully delineate the role of miR-15a in adipocyte proliferation in humans.

In summary, to date studies have identified several candidate miRNAs which can accelerate or inhibit pre-adipocyte differentiation (Table **2**). These miRNAs may provide promising candidates to design anti-obesity drugs to control fat cell development. However, it remains important to examine whether miRNAs which regulate adipocyte differentiation *in-vitro* are dysregulated in human obesity *in-vivo*. Another source of pre-adipocytes which could increase mature adipocyte numbers and hence contribute to fat mass is multipotent MSCs (Fig. **2**).

## miRNAs AND MULTIPOTENT MESENCHYMAL STEM CELLS DURING ADIPOGENESIS

Multipotent MSCs are fibroblast-like cells present in multiple tissues such as bone marrow but with potential to differentiate into diverse cell types including adipocytes, osteocytes and chondrocytes [73]. Unfortunately, multipotent MSCs are difficult to define due to the lack of established cellular markers [54]. Furthermore, the factors governing the adipogenic lineage commitment of multipotent MSCs have not been well characterized [74]. Adipogenic lineage commitment can be activated by developmental factors such as BMP (Bone-morphogenic protein) resulting in the development of an adipo-fibroblast type cell. Mesenchymal stem cell derived pre-adipocytes can subsequently undergo terminal differentiation into mature adipocytes (Fig. **2**). 

Bone marrow is a rich source of multipotent MSCs, the vascular stroma of adipose tissue also harbors multipotent MSCs [73]. Studies in the last two years have reported miRNA expression is altered during adipogenic lineage commitment of multipotent MSCs [56, 64, 68, 69, 75-78]. Upregulation of adipogenic lineage commitment in the multipotent MSC population could contribute to adipocyte hyperplasia in obesity. However to date the contribution of multipotent MSCs to increased adipose tissue mass in obesity is largely unknown.

### Mouse Studies

Opposing effects of miR-24 and miR-31 have been reported in the C3H10T1/2 multipotent mouse embryonic stem cell line treated with BMP2 to induce adipogenic differentiation [64]. Neither miR-24 nor miR-31 alone was sufficient to alter development of multipotent MSCs to mature adipocytes, but in the presence of BMP2, miR-24 overexpression accelerated mature adipocyte marker expression, while miR-31 overexpression suppressed the adipogenic markers PPARG, CEBPA and aP2 [64]. A putative miR-31 binding site in the 3’UTR of CEBPA was experimentally validated [64]. CEBPA is an enhancer binding protein which can bind to the promoter of leptin. Therefore, miR-31 downregulation could indirectly suppress leptin *via *translational repression of CEBPA during adipogenic lineage commitment of MSCs [64].

Leptin is a well-established adipose hormone which regulates appetite and fat storage [79]. However, leptin mimics developed as anti-obesity agents have been unsuccessful to date, due to leptin resistance in obese humans [3]. Interestingly, according to the miRNA target finding algorithm TargetScan the 3’UTR of leptin harbors putative miRNA binding sites for miR-9, miR-490, miR-29 family, miR-27 family and miR-128 [44]. In addition, the 3’UTR of the leptin receptor gene contains putative binding sites for the miR-200 family and the miR-30 family [44]. However, these putative miRNA binding sites remain to be experimentally validated. In future studies it may be worthwhile investigating whether these miRNAs can modulate leptin resistance and hence the efficacy of leptin-associated anti-obesity drugs.

Further studies in mouse ST2 mesenchymal stem cells showed that expression of the mammalian miRNA cluster consisting of miR-141, miR-200a, miR-200b, miR-200c and miR-429 is upregulated during adipogenic differentiation [80]. This miRNA cluster has been shown to be a negative regulator of WNT signaling which blocks adipogenic differentiation in multipotent MSCs [80]. Further work in the ST2 mesenchymal cell-line demonstrated overexpression of miR-378/378* could increase lipid droplet size independently of C/EBP isoforms and PPARγ1 which are known to stimulate increases in lipid droplet size [56]. Conversely, knockdown of miR-378/278* decreased triglyceride accumulation [56]. Interestingly, miR-378/378* is encoded within the intron of PGC-1β and is highly induced during differentiation of mouse 3T3-L1 pre-adipocytes and mouse pre-adipocytes [56]. These findings suggest miR-378 inhibitors could potentially suppress differentiation of both multi-potent MSCs and pre-adipocytes and hence reduce the pool of mature lipid storing adipocytes.

### Human Studies

Several studies have reported screening miRNA changes during adipogenic differentiation of human multipotent mesenchymal stem cells [54, 60, 66, 69, 75, 77]. Unlike mouse MSCs miR-31 and miR-24 were not reported to be altered during adipogenic differentiation of human multipotent MSCs [75, 76]. Twenty miRNAs were identified as upregulated during adipogenic differentiation of human multipotent MSCs, but only one miRNA was downregulated [75]. Leukemia inhibitor factor (LIF) mRNA was found to be a direct target of two miRNAs, miR-199 and miR-346. A luciferase reporter construct containing the 3’UTR of LIF showed miR-199 and miR-346 could both bind putative target sites in LIF [75]. LIF is a pleiotropic cytokine implicated in the maintenance of stem cells [75].

Another recent study in human multi-potent MSCs reported miR-21 transiently increased during early adipogenic differentiation [76]. Overexpression of miR-21 in human multi-potent MSCs promoted adipogenic differentiation [76]. TGFBR2 was demonstrated to be a bone-fide miR-21 target. Furthermore, miR-21 was found to alter SMAD3 phosphorylation, and involved in TGF-β signaling which is known to inhibit adipocyte differentiation [76].

miRNAs may also inhibit adipogenesis in human multi-potent MSCs. For example, miR-138 is reported to be downregulated during adipogenic differentiation [77]. In addition, overexpression of miR-138 in human multi-potent MSCs during adipogenesis could effectively reduce lipid droplet accumulation [77]. MiR-138 has been demonstrated to target the 3’UTR of EID-1, an interacting inhibitor of differentiation that can interact with SHP, an endogenous enhancer of adipogenic PPARγ2 [77]. Therefore miR-138 appears to indirectly regulate PPARγ, an established  transcription factor driving adipogenic gene expression in human MSCs [11].

Studies in human and mouse MSCs have demonstrated miRNAs are important players in adipogenic lineage commitment which suggests MSCs have the potential to contribute to adipocyte hyperplasia in obesity. However, to date no studies have established whether dysregulation of multipotent MSCs occurs with increased adipose tissue mass and obesity.

## miRNAs IN BROWN ADIPOCYTES

Adipose tissue contains both white and brown adipocytes. Recent findings suggest brown adipocytes are derived from distinct precursors closely related to skeletal muscle [81]. Brown adipocytes have a phenotype distinct from white adipocytes characterized by high energy expenditure rather than energy storage [82]. Brown adipose tissue is present in mice and recent findings suggest active brown adipose tissue may be present in humans [83, 84]. Brown adipose tissue appears to be negatively associated with BMI and body fat [83, 84]. Interestingly, miR-455 which is expressed at low levels in white preadipocytes and white mature adipocytes is reported to be upregulated during brown pre-adipocyte differentiation [85]. In addition, miR-1, miR-133a and miR-206 which are highly expressed in skeletal muscle [86] are reported to be absent from white adipocytes but are expressed in brown pre-adipocytes and mature adipocytes [85]. Further studies delineating miRNAs differentially regulated in brown adipocytes and miRNA targets in brown adipocytes could identify useful therapeutic targets to treat obesity. In future it may be worthwhile investigating whether transfecting brown adipocyte miRNAs into white adipocytes can shift cells towards an energy consuming brown adipocyte phenotype.

## EXTRACELLULAR FACTORS CAN REGULATE miRNAs IN ADIPOCYTES

### Cytokines

Obesity is associated with inflammatory cytokine release from adipose tissue and elevated inflammatory cytokine levels including TNF-α and IL-6 [2]. Chronic inflammation alters miRNA levels in immune cells [87], but few studies have examined the relationship between chronic inflammation and miRNA levels in adipocytes. TNF-α release from adipocytes has been reported to impair pre-adipocyte differentiation in obese subjects and hence contribute to lipid deposition in liver and skeletal muscle [15]. However, limited data exists on the effects of TNF-α on miRNA expression in differentiated adipocytes. TNF-α treatment of differentiated adipocytes downregulated miR-103 and miR-143 expression, although the mechanism responsible remains unknown [55].

Release of inflammatory cytokines from adipose tissue attracts macrophages to adipose tissue but may also increase insulin resistance in peripheral tissues [11]. A study comparing miRNA expression in human subcutaneous and omental (visceral) adipose tissue found negative correlations between miR-99a, miR-325 and IL-6 concentration [88]. In addition, adiponectin concentration was inversely correlated with miR-181a expression [88]. However, the aforementioned study included a small group of Type 2 diabetes patients. Future studies are needed to establish the mechanism linking TNF-α, IL-6 and other cytokines to adipocyte miRNA expression.

### Glucose

Incubation of 3T3-L1 adipocyte cells in glucose is reported to upregulate miR-29 and increase insulin resistance [89]. Another study in 3T3-L1 adipocytes confirmed miR-29 was induced by exposure to high extracellular glucose, in addition miR-27a and miR-222 were also found to respond to extracellular glucose concentration [90]. A further study reported miR-320 along with fifty other miRNAs was upregulated in response to hyperglycemia and hyperinsulinemia in 3T3-L1 adipocytes [91]. Inhibition of miR-320 in insulin resistance 3T3-L1 adipocytes was found to improve insulin sensitivity and insulin-stimulated glucose uptake *via *modulation of p85 expression, phosphorylation of Akt and GLUT4 protein levels [91].

It is unknown whether glucose modulates adipose miRNA expression *in-vivo*. In human adipocytes glycosylated haemoglobin (HbA1c), a marker of long term hyperglycemia, was negatively correlated with miR-17-5p and miR-134 expression, but positively correlated with miR-181a expression [88]. Only miR-132 was associated with fasting glucose levels in mature human adipocytes [88]. Again these findings require further confirmation in an experimental model. These studies suggest adipose miRNA expression may respond to changes in macronutrient availability.

### High-Fat Diet 

There is limited knowledge on how diet influences miRNA expression in adipose tissue. Experiments in 3T3-L1 cells and primary adipocytes suggest changes in miRNA expression may occur in response to nutrient availability [89, 91]. For example, miR-143 was reported to be upregulated in mesenteric fat of mice fed a high-fat diet for 8 weeks [62]. The upregulation of miR-143 in mesenteric fat was associated with body weight, plasma leptin concentration, PPARγ mRNA and aP2 mRNA levels [62]. In another study of high-fat diet fed mice, pri-miR-27a was reported to be downregulated in mature adipocytes [70], although one caveat is that pri-miRNA levels are not always consistent with functional mature miRNA levels [37]. Nevertheless the downregulation of pri-miR-27a in mice consuming a high-fat diet is consistent with the anti-adipogenic effect of miR-27a reported in 3T3-L1 cells [70]. Given the growing number of adipogenic and anti-adipogenic miRNAs identified, it is highly plausible these miRNAs will also respond to dietary changes.

Some studies have shown nutrient supplementation can reduce the adverse consequences of a high-fat diet and also coincidently modulate miRNA expression in adipose tissue [92, 93]. In future studies it will be interesting to determine whether miRNA inhibitors or mimics can block the development of obesity in high-fat diet fed mice. Furthermore, it will be interesting to determine whether current dietary compounds known to suppress diet-induced obesity also influence adipose miRNA expression.

## miRNAs ALTERED IN MAMMALIAN OBESEADIPOSE TISSUE

In adipose tissue from obese mouse models and obese humans several miRNA profiling studies have identified miRNAs associated with obesity [9, 55, 66, 88]. The findings of these studies are summarized in Table **1**. In adipose tissue of obese mice increased miR-143 expression is associated with parallel alterations in PPARγ and aP24 which are markers of adipocyte differentiation [62], although an earlier study reported downregulation of miR-143 in obese mice [55]. However, the latter study used the epididymal fat pad [55] rather than the mesenteric fat pad [62] which may explain the lack of concordance between studies.

In three murine models of obesity including leptin deficient ob/ob mice, leptin receptor deficient db/db mice and KKAy44 mice, miR-335 was found to be upregulated [63]. During adipocyte differentiation miR-335 is strongly induced, suggesting miR-335 may play a role increasing the mature adipocyte population [63]. Despite strong correlations between fat weight and miR-335 expression in obese mice, there are no experimentally validated targets of miR-335 in adipocytes. To date miR-335 has not been identified in miRNA profiling of adipose tissue from obese humans (Table **1**).

In human subcutaneous adipose tissue overexpression of miR-519d was reported to be associated with severe obesity [9]. MiR-519d was demonstrated to bind to the 3’UTR of PPARα. Despite PPARα mRNA being highly expressed in obese subjects, PPARα protein was undetectable compared to controls [9]. The discrepancy between mRNA and protein expression indicated a post-transcriptional mechanism may be regulating PPARα protein. During adipogenesis miR-519d is stimulated in a dose-dependent manner, which suggests miR-519d may be a factor in adipocyte hypertrophy and increased adipose tissue mass in human obesity [9]. No previous studies have reported miR-519d is differentially expressed in obesity or during adipogenic differentiation (Table **1**). One possible explanation may be because biopsies from adipose tissue can consist of heterogeneous cell types such as, pre-adipocytes, mature-adipocytes, macrophages and multi-potent MSCs. Hence the diversity of cell types could confound interpretation of miRNA expression in obese adipose tissue across different studies.

Also in subcutaneous adipose tissue from obese subjects miR-150 was reported to be upregulated and miR-659 was reported to be downregulated [9]. PPARGC1α is a predicted target of miR-150, while IRS2 and CPT1α are predicted targets of miR-659 according to TargetScan [44]. Unfortunately, miR-150 and miR-659 target protein levels were found to be unchanged in obese adipose tissue [9], although this finding does not rule out the possibility miR-150 and miR-659 may act on alternative targets which remain to be identified.

In future miRNA profiling studies on obese adipose tissue, it will be important to consider differences between fat depots. A recent study reported omental and subcutaneous adipose tissue appear to have unique miRNA expression profiles [88]. These findings are in-line with past studies indicating differences in gene expression between subcutaneous and visceral fat depots, and increased production of inflammatory cytokines in visceral fat depots [11]. Furthermore, fat cell development appears to differ between fat depots, for example adipocyte hypertrophy was found in abdominal fat depots while adipocyte hyperplasia was observed in femoral fat depots [94]. Many additional miRNAs identified in these miRNA profiling studies on human obese adipose tissue remain to be investigated further [9, 66].

## CIRCULATING miRNAs

In the past year circulating miRNAs have generated much research interest as novel diagnostic disease markers [8]. Distinct serum and plasma miRNA signatures have been reported in cancer [95] and other chronic medical conditions. miRNAs in serum and plasma although usually expressed at low levels are detectable by real-time quantitative PCR due to the high sensitivity of this method to detect mature miRNA expression [96]. Circulating miRNAs appear not to be simply benign disease biomarkers. For example, extracellular miRNAs appear to be secreted in small membrane vesicles called exosomes and hence protect miRNAs from degradation [97]. It also appears that tumor-suppressive miRNAs can be transported to recipient cells and cause gene target silencing in recipient cells [97]. However, the mechanism responsible for secretion and transport or miRNAs has not been well characterized. To date no studies have examined whether a distinct plasma miRNA signature is associated with obesity.

## miRNA BASED THERAPEUTIC APPROACHES FOR TARGETING OBESITY

Antagomirs have been used successfully in mice to downregulate miR-122 [98]. Antagomirs are anti-sense oligonucleotide sequences conjugated with cholesterol which can be injected into mice tail veins and inhibit specific miRNAs in liver [98]. In non-human primates systematic administration of LNA anti-miRs against miR-122 was reported to result in a dose dependent improvement of plasma cholesterol with no indication of hepatic toxicity [99]. Currently, Phase II trials are underway to assess the safety and tolerability of SPC3649, a miR-122 targeted drug, for treatment of Hepatitis C virus (HCV) infection in humans [100]. To date there are no known miRNA therapeutics designed to reduce fat mass in obesity, but clearly the technology to inhibit miRNAs in different tissues is advancing rapidly.

In a mouse model of obesity and diabetes a molecular autoregulatory system was developed to target the BDNF (brain-derived neurotrophic factor) gene which is involved in the regulation of energy balance in the hypothalamus [101]. The autoregulatory system consisted of a single recombinant adeno-virus vector harboring two expression cassettes containing sequences for BDNF and the sequence of a specific miRNA targeting BDNF [101]. The miRNA cassette was controlled by a promoter agouti-related protein which was sensitive to BDNF changes [101]. In mice as body weight decreased, the promoter in the miRNA cassette was activated inhibiting transgene expression of BDNF [101]. The autoregulatory approach was able to maintain weight loss, thus may be a plausible technique for long-term treatment of obesity [101].

## CONCLUSIONS

miRNA profiling studies have identified miRNAs involved in adipogenesis and associated with obesity, but the challenge remains to determine how these miRNAs are regulated in adipose tissue. Therefore, it would be worthwhile to investigate if there are parallel changes in miRNA transcription, biogenesis and degradation which can explain the dysregulated miRNAs observed in obesity. Furthermore, the role of other extracellular stresses and nutrient availability regulating obesity associated miRNAs remains unknown. From a clinical point of view with the recent discovery miRNAs are secreted into extracellular fluids, it will be important to establish whether a plasma miRNA profile can distinguish healthy and obese subjects. Potentially plasma miRNA profiles could be used by clinicians for obesity management and to track the efficacy of miRNA based therapeutics. Finally, further identification and characterization of miRNAs associated with adipogenesis and obesity should provide a new generation of therapeutic targets which will help facilitate the development of new anti-obesity treatments.

## Figures and Tables

**Fig. (1):**
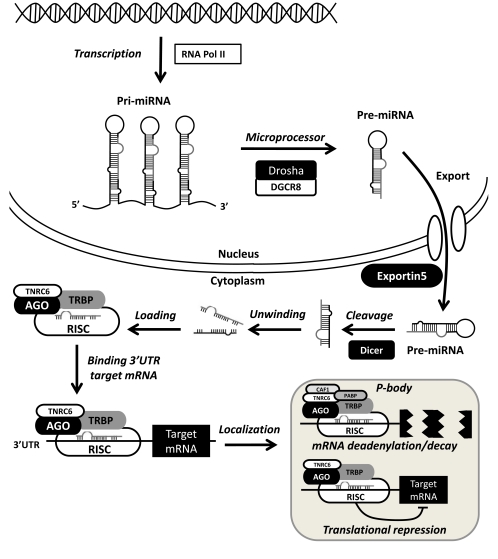
miRNA biogenesis pathway. miRNAs are transcribed by RNA polymerase II resulting in pri-miRNA transcripts. Pri-miRNAs are processed by a microprocessor complex including Drosha and DGCR8 leaving Pre-miRNAs which are exported from the nucleus *via* Exportin5. Pre-miRNAs are processed by Dicer, the mature miRNA is loaded in the RISC complex. Mature miRNAs bind the 3’UTRs of target mRNAs and localize to P-bodies. In P-bodies target mRNAs are deadenylated and degraded *via* CAF1 and PABP or translationally repressed.

**Table 1:** Mammalian miRNAs Associated with Adipogenesis and Obesity

miRNA	Function	Experiment System	Species	Targets	References

Let-7	↑ Adipogenesis	3T3-L1, Pre-ad, MSC,	H/M	HMGA2	[56, 58-61, 75]
	↓ Adipose tissue	Ob adipose tissue	H	E2F6	[9, 66]
		Ob adipocytes		CDC34	

miR-15a	↑↓ Adipogenesis	3T3-L1	M	DLK1	[61, 72]
	↑ Adipose tissue	Ob adipose tissue	H		[9]

miR-17/92	↑ Adipogenesis	3T3-L1	M	RB2	[57, 68]
				p130	

miR-21	↑↓ Adipogenesis	3T3-L1, MSC	H/M	TGFBR2	[56, 65, 76]

miR-24	↑ Adipogenesis		M		[64]

miR-27	↓ Adipogenesis	3T3-L1, MSC	H/M	PPARG	[59, 68-70]
	↓ Adipose tissue	Ob adipocytes	H/M		[68, 70]

miR-31	↑↓ Adipogenesis	3T3-L1, Pre-Ad	H/M/R	CEBPA	[56, 64, 78]
	↓ Adipose tissue	Ob adipocytes			[66]

miR-103	↑↓ Adipogenesis	3T3-L1, Pre-ad, MSC,	H/M	PDK1	[55, 56, 59-61, 65, 75]
	↓ Adipose tissue	Ob adipose tissue	M	WNT3A	[55]

miR-107	↑ Adipogenesis	3T3-L1, Pre-ad, MSC,	H/M		[55, 56, 59, 60, 75]
	↓ Adipose tissue	Ob adipose tissue	M		[55]

miR-125b	↑↓ Adipogenesis	3T3-L1, Pre-ad,	H/M		[55, 56, 66]
	↑↓ Adipose tissue	Ob adipose tissue	H/M		[55, 66]
		Ob adipocytes			

miR-138	↓ Adipogenesis	MSC	H	EID1	[77]

miR-143	↑ Adipogenesis	3T3-L1, Pre-ad, MSC	H/M	ERK5	[55, 60, 61, 64, 75]
	↑↓ Adipose tissue	Ob adipose tissue	H/M		[55, 62]

miR-150	↓ Adipogenesis	3T3-L1	M		[56]
	↑ Adipose tissue	Ob adipose tissue	H		[9]

miR-200	↑ Adipogenesis	MSC	M		[80]

miR-210	↑ Adipogenesis	3T3-L1	M	TCF7L2	[56, 59, 65]
	↑ Adipose tissue	Ob adipocytes	H		[66]

miR-221	↓ Adipogenesis	3T3-L1, Pre-ad	M		[55]
	↑ Adipose tissue	Ob adipose tissue	H/M		[55, 66]

miR-222	↓ Adipogenesis	3T3-L1, Pre-ad	M		[55]
	↑ Adipose tissue	Ob adipose tissue	M		[55]

miR-326	↓ Adipogenesis	Pre-ad	R		[78]
	↑ Adipose tissue	Ob adipose tissue	H		[66]

miR-355	↑ Adipogenesis	3T3-L1, MSC	H/M	-	[59, 63, 75]
	↑ Adipose tissue	Ob adipose tissue	M		[63]

miR-378	↑ Adipogenesis	3T3-L1, MSC	M		[56]

miR-448	↓ Adipogenesis	3T3-L1	M	KLF5	[71]

miR-519d	↑ Adipose tissue	Ob adipose tissue	H	PPARα	[9]

Pre-ad – Pre-adipocytes; Ob – Obese; MSC – Multipotent mesenchymal stem cells; H – Human; M – Mouse; R – Rat.

**Table 2:** miRNA Microarray Platforms Used in Adipogenesis and Obesity Studies

Model	Platform	References

3T3-L1	Microfluidic biochip (LC Sciences)	[55]
	Invitrogen Ncode miRNA array	[57]
	Ambion miRNA probes/Nexterion E slides	[56]
	Agilent miRNA array	[59]
	Ambion mirVana probes/Custom slides	[65]

Multi-potent MSCs	Exiqon miRCURY array	[78]

Primary Adipocytes	Custom oligo/Codelink slides	[60]

Adipose Tissue	Microfluidic biochip (LC Sciences)	[55]
	Taqman miRNA assay human panel	[88]
	Agilent miRNA array	[66]
	Exiqon miRCURY LNA miRNA array	[9]
